# Modeling of spatial pattern and influencing factors of cultivated land quality in Henan Province based on spatial big data

**DOI:** 10.1371/journal.pone.0265613

**Published:** 2022-04-08

**Authors:** Hua Wang, Yuxin Zhu, Jinghao Wang, Hubiao Han, Jiqiang Niu, Xueye Chen

**Affiliations:** 1 School of Computer and Communication Engineering, Zhengzhou University of Light Industry, Zhengzhou, China; 2 Key Laboratory for Synergistic Prevention of Water and Soil Environmental Pollution, Xinyang Normal University, Xinyang, China; 3 Key Laboratory of Urban Land Resources Monitoring and Simulation, Ministry of Natural Resources, Shenzhen, China; Universidade Federal de Uberlandia, BRAZIL

## Abstract

The quality of cultivated land determines the production capacity of cultivated land and the level of regional development, and also directly affects the food security and ecological safety of the country. This paper starts from the perspective of spatial pattern of cultivated land quality and uses spatial autocorrelation analysis to study the spatial aggregation characteristics and differences of cultivated land quality in Henan Province at the county level scale, and also uses bivariate spatial autocorrelation to analyze the influence of neighboring influences on the quality of cultivated land in the target area. The spatial autoregressive model was used to further analyze the driving factors affecting the quality of cultivated land, and the influence of cultivated land area index was coupled in the process of rating analysis, which was finally used as a basis to propose more precise measures for the protection of cultivated land zoning. The results show that: (1) The quality of cultivated land in Henan Province has a strong spatial correlation (global Moran’s I≈0.710) and shows an obvious aggregation pattern in spatial distribution; positive correlation types (high-high and low-low) are concentrated in north-central and western mountainous areas of Henan Province, respectively; negative correlation types are discrete. The negative correlation types are distributed in a discrete manner. (2) The bivariate spatial autocorrelation results show that Slope (Moran’s I≈-0.505), Irrigation guarantee rate (IGR, 0.354), Urbanization rate (-0.255), Total agricultural machinery power (TAMP, 0.331) and Pesticide use (0.214) are the main influencing factors. (3) According to the absolute values of the regression coefficients, it can be seen that the magnitude of the influence of different factors on the quality of cultivated land is: Slope (0.089) >IGR (0.025) > Urbanization rate (0.002) > TAMP (0.001) > Pesticide use (1.96e-006). (4) Based on the spatial pattern presented by the spatial autocorrelation results, we proposed corresponding protection zoning measures to provide more scientific reference decisions and technical support for the implementation of refined cultivated land management in Henan Province.

## Introduction

Cultivated land is the material basis for human survival and development. And the quality of cultivated land is related to the level of regional utilization and productivity of cultivated land, which has a direct impact on food security and ecological security [1]. The quality of cultivated land varies greatly across regions due to demographic, economic and natural factors [2], but it is not only influenced by ecological environment, soil nutrients and its combination, but also closely related to the uniformity and complexity of its own spatial distribution [3]. Therefore, studying the spatial aggregation characteristics and regularity of cultivated land quality can not only help to better understand the current situation of cultivated land utilization in the region [4], but also carry out the work of cultivated land protection, so as to improve the quality of cultivated land and ensure the rational use of resources [5].

In recent years, the research on the spatial pattern of cultivated land quality has gradually attracted the attention and exploration of many scholars at home and abroad [6]. In terms of research methods, most scholars used autocorrelation analysis, kriging interpolation and landscape ecology [7] and other technical means to study the spatial pattern of cultivated land [8]. Among them, Kriging interpolation is mainly used to describe and model the spatial pattern of cultivated land quality [9], and landscape ecology is often used to study the change trend of spatial pattern of cultivated land [10]. Both of these ignore the impact of spatial heterogeneity on the quality of cultivated land. Spatial autocorrelation technology has comprehensively considered the differences in the spatial distribution of cultivated land quality [11], and can visualize spatial data while analyzing the spatial aggregation law of cultivated land quality. Therefore, it has been widely used in spatial research in recent years [12]. In terms of research objects, domestic research on cultivated quality is mainly focused on the analysis of factors affecting cultivated quality and cultivated quality monitoring or rating, but relatively few studies on the spatial characteristics of cultivated quality [13], and most studies do not further analyze the driving factors affecting cultivated quality. In addition, existing studies mainly favor a prefecture-level city or county as the target area for analysis [14], and most of them only consider the natural quality attributes in the analysis process, ignoring the impact of cultivated land area on the comprehensive quality rating, which cannot comprehensively grasp the spatial distribution characteristics of cultivated land quality in macro-regions and is not conducive to proposing more scientific and reasonable cultivated land utilization and protection measures [15].

Therefore, this paper chooses Henan Province, the most important agricultural, livestock and food production base in China, to analyze the spatial differences and distribution rule of cultivated land quality in this area. This research not only provides national food security, but also ensures sustainable development of the region. In the research process, this paper starts from the perspective of spatial correlation, then uses spatial autocorrelation, spatial autoregression and other methods to comprehensively analyze the spatial aggregation characteristics and differences of cultivated land quality in Henan Province at the county level. At the same time, the influence of area attributes is taken into account in the process of rating the quality of cultivated land to avoid a situation where cultivated land is of high grade but too small in size [16], using data normalization to obtain cultivated land quality grade coupled with area attributes(CLQGCA). Then, this paper uses bivariate spatial autocorrelation to analyze the influence of neighboring influences on the quality of cultivated land in the target area [17], and uses the spatial autoregressive model to further analyze the main drivers of the influence on cultivated land quality in Henan Province. It will ensure the objectivity and scientificity of the cultivated land quality rating results, and provide more scientific and reasonable decision-making reference for the implementation of targeted cultivated land protection and refined cultivated land management in Henan Province.

## Data and research methodology

### Data

#### Overview of the study area

**Fig 1:**
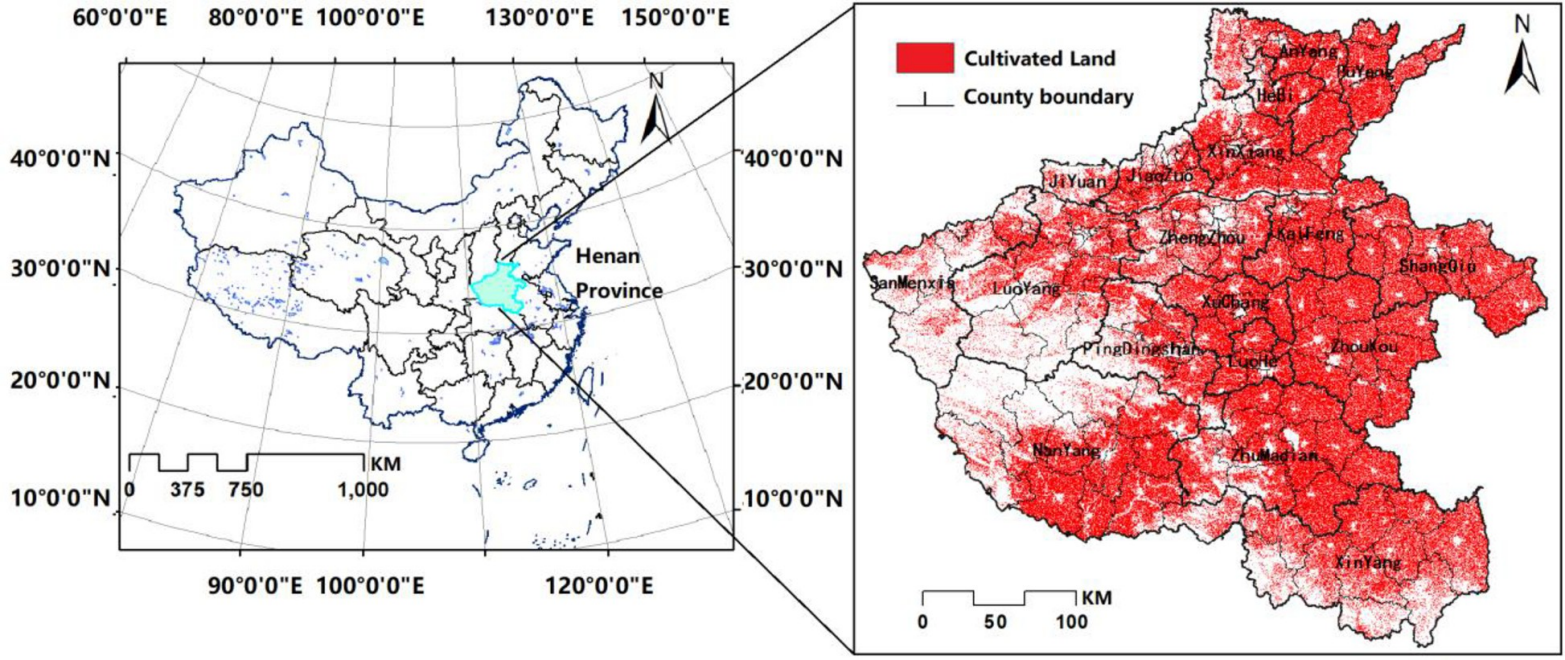
Location map of Henan Province.

Henan province is located in the central region of China, with the boundary between 31°23’-36°22’ north latitude and 110°21’-116°39’ east longitude (The specific location and distribution of arable land is shown in Fig 1). It is an important transportation hub and one of the largest grain bases in China. Henan Province has a total area of 167,000㎢, of which mountains as well as hills account for about 26% and 18% of the total area respectively, mostly located in the western region, where forestry and animal husbandry are mainly developed; plains account for about 56%, mostly located in the central and eastern region, so most of the province’s cultivated land is gathered there. The province’s cultivated land covers an area of 81122.26 ㎢, ranking third in the country, with about 1.27 mu of cultivated land per capita. In 2018, the total grain output in Henan Province was about 66.49 million tons, ranking second in the country.

#### Data preprocessing

This paper takes the quality of cultivated land in 159 urban areas of Henan Province in 2018 as the research object, details of the sources of the underlying data are shown in Table 1. Based on the standard of "Cultivated Land Quality Grade", the quality of cuitivated land is divided into 15 grades.

**Table 1:** Description of data sources.

No.	Data	Description of Data Source	Department of Data Source
**1**	Natural quality grade, utilization grade and economic grade of cultivated land	Database of the 2018 Cultivated Land Quality Grade Update Project in Henan Province	Natural Resources Planning Bureau of Henan Province
**2**	Data on slope, IGR,TAMP and other influencing factors in each county	cultivated land quality update evaluation results and County Statistical Yearbook(2019)	Agriculture Bureau
**3**	Town bound boundaries	Industrial and tourism development planning	Development and Reform Commission

Data pre-processing consists of two main modules, the conversion of information files into standardized geographic information and the processing of map data. First, ArcGIS 10.2 was used to unify the coordinate system of all the above data and drawing data, so as to ensure data standardization and similarity of properties within the study unit. To avoid too fragmented results of spatial autocorrelation analysis when identification is carried out on the basis of cultivated land patches, the calculation unit was transformed from townships to counties through the area-weighted average method in this study, and coupled the normalized results of the area index in the process of rating the quality of cultivated land. In turn, the spatial autocorrelation analysis and the spatial autoregressive model were completed.

### Research methodology

#### Area weighting method and data normalization

Determination of spatial weights is the basis for spatial correlation analysis, through the spatial statistical analysis of the adjacency of cultivated land patches in the study area. This paper selects the Queen adjacency principle to determine the spatial weights and constructs the corresponding spatial weight matrix [18]. The specific formula for the area-weighted method is as follows:

$$Y_{i}=\frac{\sum_{i\min}^{i\max}i\times Si}{\sum S_{i}}$$ (1)

where Y_i_ denotes the average cultivated quality of the target county, *i* denotes the cultivated quality index of the corresponding township, S_i_ represents the corresponding cultivated area of township *i*, and the denominator is the sum of the cultivated areas of all the townships under the study, that the total cultivated area of the county.

Cultivated land quality indices and area attributes are important spatial attributes of cultivated land, and most of the existing studies often ignoring the influence of cultivated land area [19]. In order to eliminate the influence of scale between indicators [20], this study normalized the natural quality of cultivated land and the area of cultivated land separately, so that they are in the same order of magnitude. The normalized results were coupled with a weight of 0.5, and then the spatial autocorrelation analysis was performed on the coupled results to ensure the scientific accuracy and objectivity of the research results. The specific formula for the data-normalization method is as follows:

$$X^{*}=\frac{x_{i}-\min}{\max-\min}$$ (2)

Where *x*_*i*_ is the corresponding natural class of each patches in the county and the area of the patches land class, *max* is the maximum value in the target data set in the county, and *min* is the minimum value.

#### Spatial autocorrelation analysis

The first law of geography proposed by Tobler(1970) has been the theoretical basis for spatial autocorrelation analysis, and cultivated land as continuous space also satisfies this law [21] that there is spatial correlation or similarity. Detecting the presence of spatial autocorrelation between things and measuring the degree of correlation depend on the global spatial autocorrelation and local spatial autocorrelation indices, respectively [22]. This paper used the most commonly used global Moran’s I to measure the spatial dependence of cultivated quality indices in each county in Henan Province, calculated as follows:

$$I=\frac{n\sum_{i=1}^{n}\sum_{j=1}^{n}wij(Xi-\bar{X})(Xj-\bar{X})}{\sum_{i=1}^{n}\sum_{j=1}^{n}wij\sum_{i=1}^{n}{\left(Xi-\bar{X}\right)}^{2}}$$ (3)

Where n represents the total number of spatially observed objects in the target area; *X*_*i*_ and *X*_*j*_ are the observations of the *i-th* and *j-th* objects in the spatial location respectively, $\bar{X}$ is the average observation of all the objects; and *W*_*ij*_ is the spatial weight matrix, and the value of *W*_*ij*_ indicates the adjacency between the *i-th* and *j-th* objects in the spatial location. The value of *W*_*ij*_ is the spatial weight matrix, and the value of *W*_*ij*_ indicates the neighborhood between *i* and *j*. When *W*_*ij*_ = 1, *i* is adjacent to *j*, and when *W*_*ij*_ = 0, *i* is not adjacent to *j*. The value of Global Moran’s I ranges from ±1, and if I>0, it indicates a positive spatial correlation; when I<0, it indicates a negative spatial correlation. If I = 0, it means random distribution of research objects.

Spatial autocorrelation analysis generally has only one variable, but studies have found that changes in aggregation of the same attribute unit are influenced by other attribute units in the neighborhood. Bivariate spatial autocorrelation can reflect the spatial aggregation relationship between two different attribute variables, indicating the spatial aggregation difference between the value of an observed variable in the region and the mean value of another variable in its neighborhood, calculated as follows: (The meanings of the letters are the same as in Eq 3)

$$I=\frac{\sum {}_{i}(\sum {}_{j}^{}w_{ij}y_{j}^{}\times x_{i})}{\sum {}_{i}x_{i}^{2}}$$ (4)

Although global spatial autocorrelation analysis can reflect whether the research objects are clustered in space, it cannot further show the location and characteristics of spatial clustering. Local spatial autocorrelation analysis can more intuitively show the spatial agglomeration of cultivated land quality in the study area [23]. In this paper, the local spatial autocorrelation is performed for each county in Henan Province, and the LSA(Local Spatial Autocorrelation) clustering map is used to analyze the spatial location of clustering or dispersion of cultivated quality in Henan Province. The calculation formula is as Formula (5), The formula for bivariate local spatial is as Formula (4).


$$I=\frac{\sum_{j=1,j\neq i}^{n}wij(xi-\bar{x})(xj-\bar{x})}{\sum_{i=1}^{n}{\left(xi-\bar{x}\right)}^{2}}$$ (5)

#### Spatial autoregression model

In analyzing the drivers affecting cultivated land quality, traditional statistical approaches rely on data being evenly distributed and independent of each other within the study area, and classical linear regression models make this assumption in advance [24]. But in reality, spatial autocorrelation prevails. The spatial autoregressive models (spatial lag and spatial error models) then modify this by incorporating spatial dependence into the regression equation [25]. Therefore, this paper selects a spatial autoregressive model to analyze the influencing factors of cultivated land quality in Henan Province. It is calculated as follows:

Y=ρW1+βX+μμ=λW2+ε (6)

Y=βX+εε=λW2ε+μ (7)

where Y is the dependent variable; X is the explanatory variable; β represents the spatial regression coefficient of the explanatory variable; μ is the error term; ε is the white noise; W1 is the spatial weight matrix reflecting the spatial trend of the dependent variable itself, and W2 is the spatial weight matrix reflecting the spatial trend of the residuals.ρis the coefficient of the spatial lag term and λ is the spatial error coefficient, both of which range from zero to one (0–1). The closer they are to 1, the more similar the values of the dependent or explanatory variables are in adjacent regions.

## Results and analysis

### Spatial distribution of cultivated land quality

**Fig 2:**
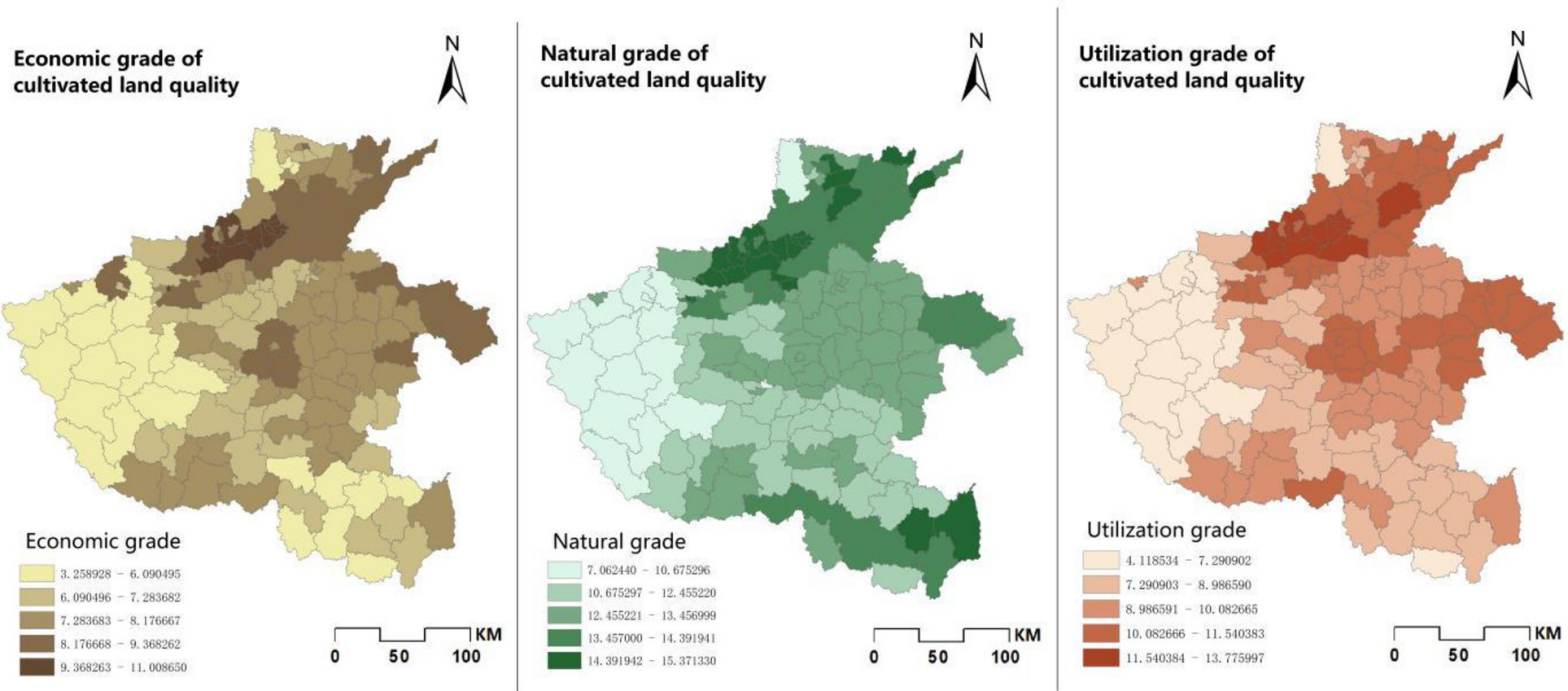
Spatial distribution of cultivated land quality in Henan Province.

Due to the diversity of landforms and climatic differences in different regions of the province, the natural quality of cultivated land in various regions is also different. In order to further observe the spatial distribution of cultivated quality, we used the natural breakpoint method to classify cultivated land quality into five levels [26]. In ArcGIS, the cultivated land quality index obtained after data preprocessing and its corresponding grades are output as a map [27]. It can be seen from the Fig 2 that the natural quality grades of cultivated land in Henan Province are mainly concentrated in 12–13. There are 55 counties with natural grades between 12–13, covering an area of 38,505㎢, accounting for 47.46% of the province’s cultivated land area. The cultivated land with higher score is mainly concentrated in the northern and central plains of Henan Province, these areas are generally fertile and have ample water resources. The cultivated land with low score is mainly concentrated in the western hilly area, which is affected by soil and terrain, etc., and the cultivated land in this area has poor natural conditions.

The utilization grade of cultivated land quality in Henan Province is predominantly grade 10–11, with a total area of 26,883㎢, accounting for 33.14% of the total cultivated land area of the province. The utilization level of cultivated land is also spatially distributed in the form of agglomeration. The cultivated land with higher utilization level is concentrated in the northern and central-eastern plains, while the low-scoring cultivated land is mostly distributed in the western hilly areas. The spatial distribution of the economic and utilization categories is similar, with high scores concentrated in the northern part of Henan Province and the central east, mainly in the 7–8 region, with an area of 31,909㎢, accounting for 39.33% of the total cultivated land, which is due to the convenience of transportation in the region and the better development of specialty products circulation and tourism, especially in cities such as Puyang, Xinxiang, Jiaozuo and Zhengzhou.

**Fig 3:**
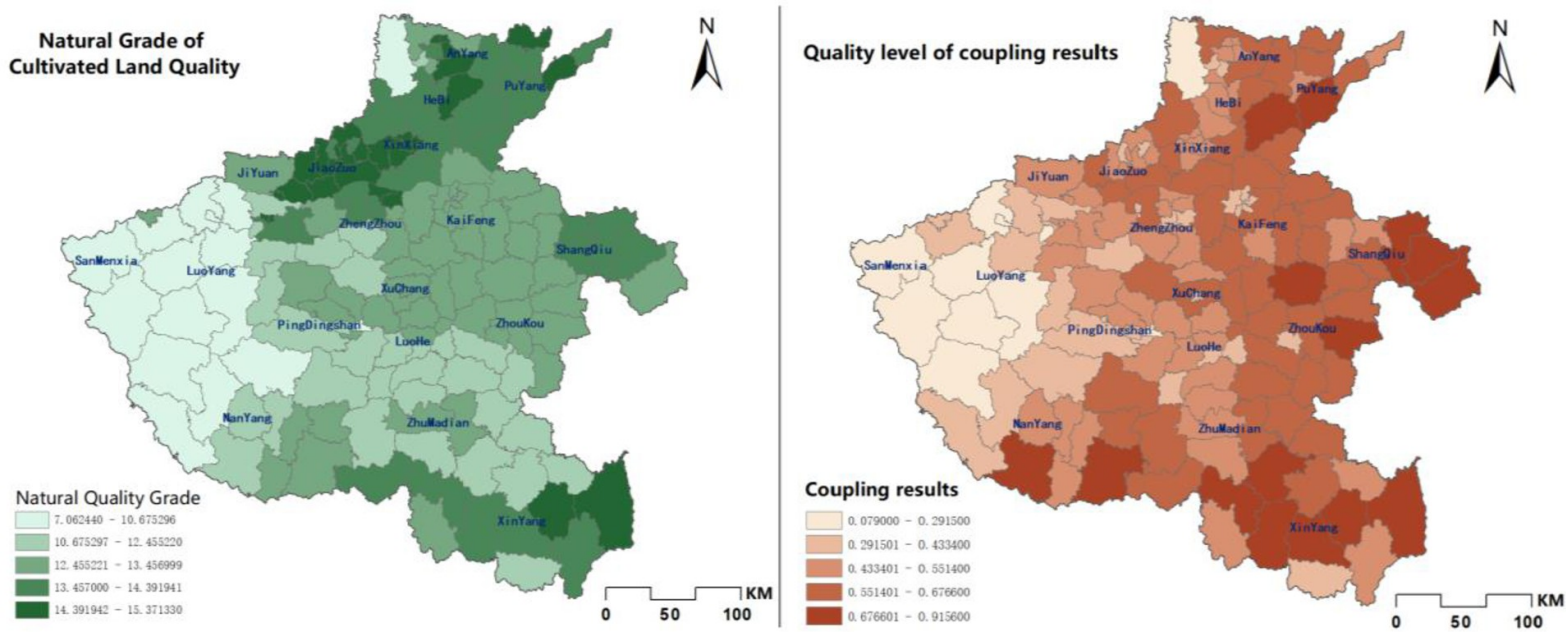
Comparison of CLQGCA with natural grade of cultivated land quality.

In general, the quality of cultivated land in Henan Province is more evenly distributed spatially. The spatial distribution of cropland quality after coupling the area attributes is also very regular (As shown in Fig 3). And cultivated land of similar natural quality is mostly distributed in clusters in the region, which is very conducive to land remediation or improvement projects according to local conditions. It also has obvious characteristics of agglomeration in spatial distribution, which is higher in the north than in the middle than in the west. It is because the central and northern plains are conducive to urban expansion and the construction of transportation routes, which drives economic development. The developed economy makes cultivated infrastructure more perfect, and due to climatic reasons, there is sufficient rainfall and heat, and abundant groundwater resources, and the quality of cultivated land in Henan Province is higher than in the west. Soils are generally fertile and the productivity of cultivated land is high, resulting in a high level of cultivated land use and natural quality in the north-central region.

### Results of spatial autocorrelation analysis

**Fig 4:**
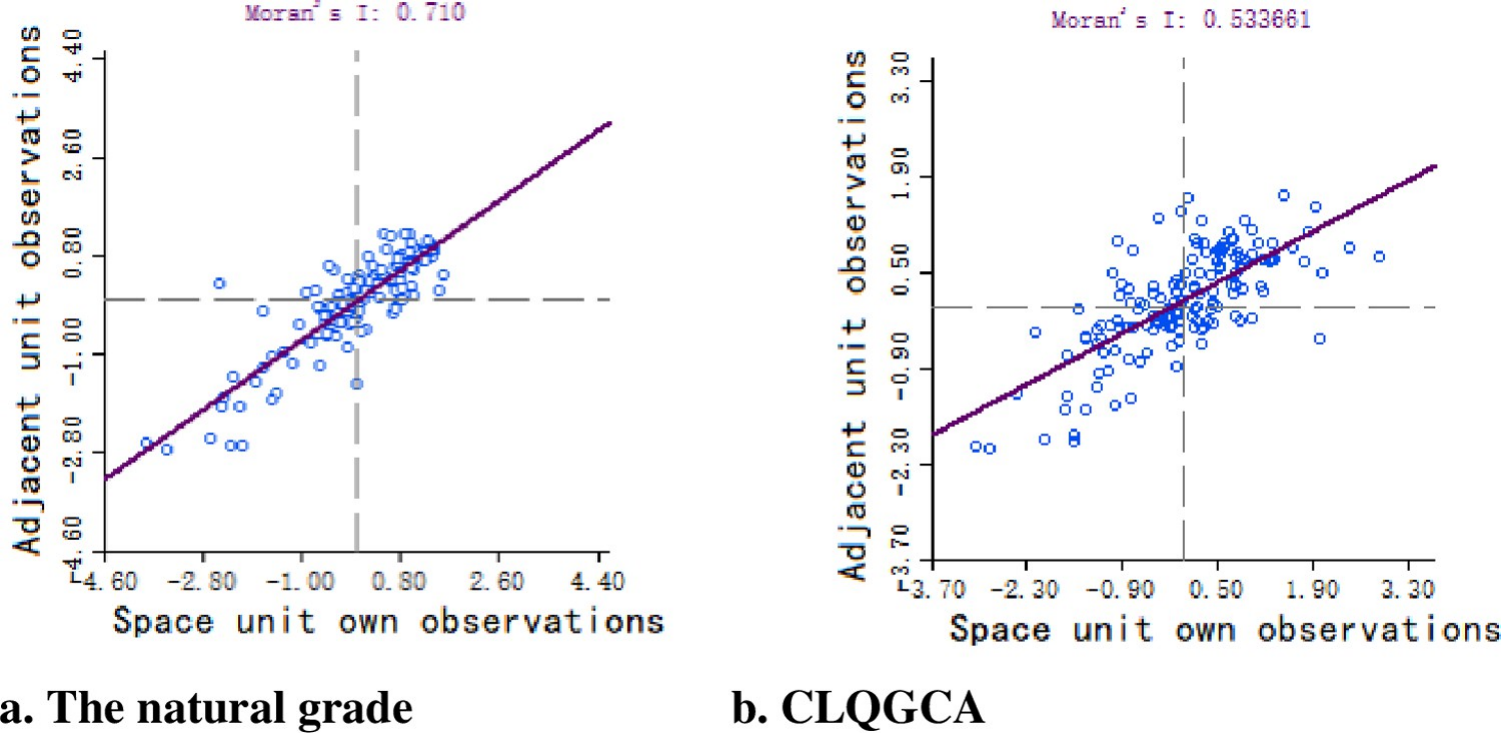
Henan province cultivated land quality index Moran scatter plot. a. The natural grade, b. CLQGCA.

This paper calculate Global Moran’s I values for spatial autocorrelation analysis, yields the Moran’s scatter plot as shown in Fig 4. From the magnitude of Moran’s I value, the quality of cultivated land in Henan Province all show more obvious positive spatial correlation [28]. The natural quality of cultivated land (Moran’s I ≈ 0.710) and the CLQGCA (Moran’s I ≈ 0.534), both results are greater than 0.5, indicating that that the quality of cultivated land has a strong spatial correlation in space. The spatial distribution of cultivated land quality is aggregated. In the figure, the horizontal axis represents the observations of the spatial cell itself, and the vertical axis represents the observations of the neighboring cells.

To further demonstrate the specific spatial aggregation location and characteristics [29], this study conducted local spatial autocorrelation analysis on the natural quality class of each county and district in the province and CLQGCA, respectively, to demonstrate the spatial aggregation characteristics of cultivated land quality in the province through LSA maps. The High-High and Low-Low types indicate that the spatial distribution of cultivated land quality has more obvious aggregation characteristics, while the Low-High and High-Low types indicate that the spatial distribution of cultivated land quality is negatively correlated, and the Not-Significance indicates that there is no significant autocorrelation.

Combining the LSA map with the results of the local spatial autocorrelation analysis in Table 2, it can be seen that there are 33 and 17 counties and districts of HH and LL types respectively, accounting for 20.75% and 10.69% of the total number of counties and districts in the province respectively, where the spatial distribution of CLQGCA is spatially positively correlated. The number of HL-type counties in the type of spatial autocorrelation is 0, and there are only three LH-type counties accounting for 1.89%; there are 106 counties with non-significant spatial autocorrelation, accounting for 66.67% of the total number of counties in the province. The spatial distribution of HH type is mainly concentrated in the northeast and southeast of Henan Province; LL type is concentrated in groups in the western. LH type cultivated land is sporadically distributed around the high score cultivated land, such as Puyang County, Xin County, etc.

**Table 2:** Types of local spatial autocorrelation and statistical summary of county in Henan province.

Autocorrelation types	cultivated land quality index		CLQGCA index	
	Number	Ratio/%	Number	Ratio/%
**High-High**	32	20.13	33	20.75
**High-Low**	0	0	0	0
**Low-High (LH)**	1	0.63	3	1.89
**Low-Low (LL)**	22	13.84	17	10.69
**non-significant**	104	65.41	106	66.67
**aggregate**	159	100.00	159	100.00

Comparing the results of local spatial autocorrelation of natural quality of cultivated land without coupled area indicators, it can be seen that some high-high types of counties located in the north became insignificant types after coupling, such as Wen County and Wuzhi County in Jiaozuo City; while counties and districts in Xinyang City, Taikang County and Xihua County in Zhoukou City, Qixi County and Lankao County in Kaifeng City turn from insignificant types into high-high type areas. This is due to the fact that the quality of cultivated land in regions such as Wen County and Wu Zhi County is high but the area of cultivated land is small, which does not allow for large-scale development of planting facilities, so the mean value is lowered after the coupled area factor. In contrast, the natural quality of cultivated land in Taikang and Xihua counties, as well as Qixian and Lankao counties, is at a medium level, but the cultivated land area is large and suitable for remediation and development of planting and construction land, so the mean value is pulled up after coupling the area index and becomes a high—high type area.

In general, the quality of cultivated land in Henan Province is spatially distributed in the form of clusters. The positive correlation between HH and LL type cultivated land is particularly obvious, and the LSA diagram shows that HH type cultivated land is gathered in the northeast and southeast of Henan Province with the trend of distribution along the river, which is due to the advantages of flat terrain, convenient transportation and abundant groundwater resources in the eastern plain, which makes the cultivated land in this area not only of high natural quality, but also with a developed economy and perfect farmland infrastructure in the region, so the input and output efficiency are both high. In contrast, LL-type cultivated land is concentrated in the western hilly areas in the form of hugging by factors such as topography and soil quality, and this area is generally inaccessible and economically backward. Under the influence of spatial polarization, the negatively correlated LH-type cultivated land is scattered around the high-score cultivated land. The positive correlation type dominates the cultivated land with spatial correlation in Henan Province status. Meanwhile, combined with the map of spatial distribution of cultivated land quality (Fig 2), it can be seen that most of the cultivated land with higher grade maintains a high consistency in spatial distribution with HH type, while most of the cultivated land with lower grade is basically consistent with the distribution of LL type area. This shows that most of the cultivated land in Henan Province has relatively similar quality attributes and spatial attributes.

### Bivariate autocorrelation results analysis

**Fig 5:**
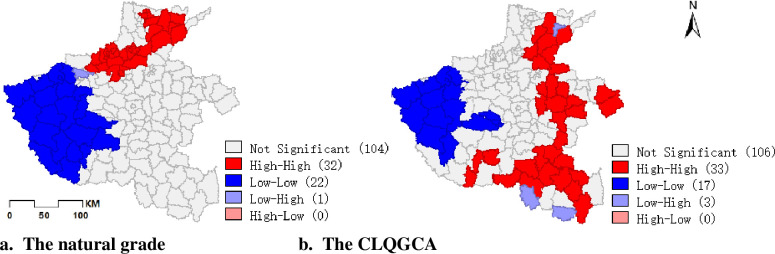
Local space autocorrelation LSA map: a) the natural level LSA map, and b) the CLQGCA LSA map.

**Fig 6:**
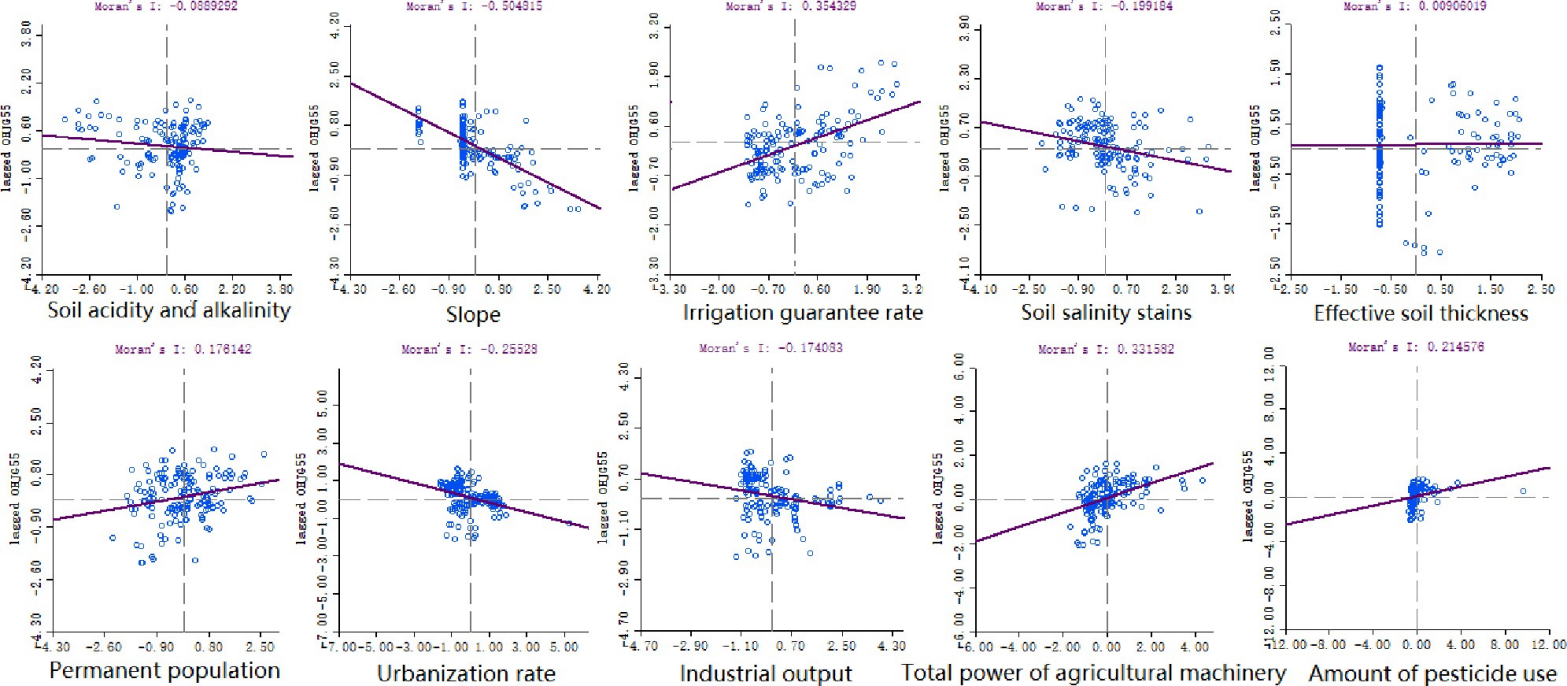
Bivariate spatial autocorrelation scatter plot.

In the actual farming process, the quality of cultivated land receives different degrees of influence from natural, human and social development factors. Based on the "Spatial and Temporal Evolution and Driving Forces of Cropland Quality in Henan Province" [30], this paper selects 10 influencing factors from the socio-economic and natural conditions categories that are crucial to the quality of cultivated land and does a bivariate spatial autocorrelation analysis with the cultivated land quality coupled with area indicators (Figs 5 and 6). Among them, the natural conditions include slope, soil acidity, IGR, effective soil thickness, and soil salinity; the socio-economic category factors include: pesticide use, urbanization rate, industrial output, population size, and TAMP.

**Fig 7:**
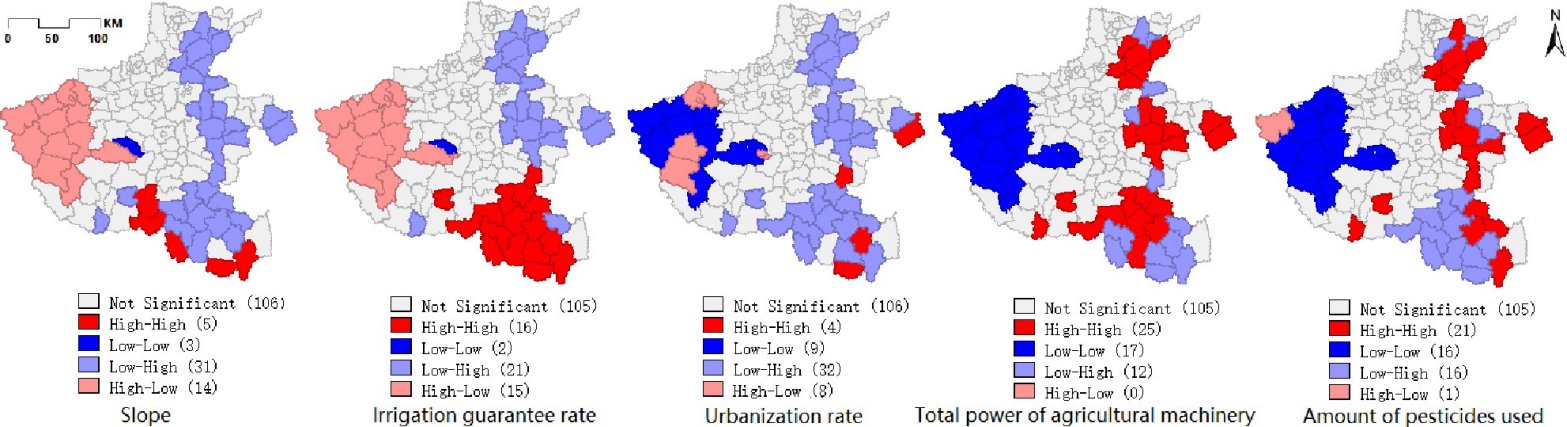
Bivariate local spatial autocorrelation LSA map.

From Fig 6, it can be seen that the factors that have a strong correlation with the CLQGCA: slope, IGR, urbanization rate, TAMP and pesticide use. The spatial correlation between slope and urbanization rate, and cultivated land quality is negative, indicating that the mean value of cultivated land quality decreases with increasing slope and urbanization rate. However, when the IGR, TAMP and pesticide use increase, the spatial correlation between cultivated land quality and itself will increase. In order to further study the specific aggregation characteristics of cultivated land quality in the region influenced by neighboring factors, bivariate local autocorrelation analysis was done for each of the five factors with strong correlation in this paper, and the results are shown in Fig 7.

The bivariate local spatial autocorrelation results show that the mean value of slope is higher in western Henan Province, while the mean values of the other four influencing factors are lower. In the central-eastern and northern parts of Henan Province, the irrigation guaranteed rate, the total power of agricultural machinery and the use of pesticides are all high, and the mean value of cultivated land quality is also high, indicating that these three influencing factors have a positive impact on the quality of cultivated land in the corresponding regional space, and the high values have a tendency to be distributed along the Yellow river. The urbanization rate is the opposite, with the lower urbanization level concentrated in the central-eastern and northern parts of Henan Province, where the quality of cultivated land is higher, indicating that the level of urbanization development is spatially opposed to the quality of cultivated land. While the western mountainous areas are relatively backward in terms of development level, the quality of arable land is still low due to geographical constraints, high slope, lack of water resources and poor conditions of agricultural facilities.

### Influence factor analysis

This study selected five influencing factors with strong spatial correlation with the quality of cultivated land, namely IGR, slope, urbanization rate, pesticide use and TAMP, as independent variables and CLQGCA as dependent variables, and constructed a spatial regression model. In this paper, we adopt these two models to analyze the influencing factors of cultivated land quality in Henan Province and compare the fit of the two models respectively.

Classical linear regression analysis based on ordinary least squares(OLS), is a necessary step before conducting spatial autoregressive analysis. In the results of this model, if other conditions are held constant, the regression coefficients indicate the extent to which the dependent variable is affected by changes in this particular independent variable; both t-Statistics and p-value are used to test the significance of the effect of the independent variable on the dependent variable, and the results are shown in Table 3.

**Table 3:** Classical model.

Impact Factors	Coefficient	Standard Error	t-Statistics	p-value
**Constant**	0.596132	0.0476252	12.5172	<0.0001
**IGR**	0.0174587	0.0139102	-1.25509	0.21136
**Slope**	-0.0786108	0.0168336	-4.66988	<0.0001
**Urbanization rate**	-0.00132278	0.000814639	-1.62377	0.10648
**Pesticide use**	5.89531e-006	5.81535e-006	1.01375	0.31230
**TPOAM**	0.0012688	0.000182707	6.94446	<0.0001

Since the traditional classical linear regression model assumes in advance that there is no spatial autocorrelation between the study data, but in practice the spatial units are all correlated, and the spatial autoregressive model modifies this by incorporating spatial dependence into the regression equation. Therefore, the spatial autoregressive model can better reflect the spatial relationship between geographical things. In addition, the OLS model results are listed in the Table 4 with the parameters used to select which spatial autoregressive model is more consistent with the objective facts.

**Table 4:** Spatial correlation test.

TEST	MI/DF	VALUE	PROB
**Moran’s I(error)**	0.3763	7.6129	0.00000
**Lagrange Multiplier(lag)**	1	29.6908	0.00000
**Robust LM(lag)**	1	0.0527	0.78856
**Lagrange Multiplier(error)**	1	19.7326	0.00000
**Robust LM(error)**	2	49.4234	0.00000

As can be seen from Table 4, the residual Moran’s I of the classical linear regression model is 0.3763 and passes the significance test, indicating the existence of spatial autocorrelation of the residuals and the introduction of a spatial autoregressive model is very necessary. However, the introduction of classical linear regression analysis is a necessary step before conducting spatial autoregressive analysis, because the results of the corresponding spatial correlation test by OLS can provide criteria for the selection of spatial autoregressive models: first discriminate the significance of Lagrange Multiplier(lag) and Lagrange Multiplier(error), if one of them is significant, use the corresponding spatial autoregressive model; if both are significant, continue to compare the values of Robust LM(lag) and Robust LM (error). In this case Robust LM(error) is more significant, so the error model should be chosen.

From the results of the regression coefficients and significance calculations in Table 5, slope, IGR, as well as urbanization rate and TAMP have more significant effects on the quality of cultivated land, with p-value < 0.01 for slope and TAMP, indicating that both pass the 1% significance test and the calculation results are closer to the actual situation. In terms of the absolute values of the regression coefficients, IGR and slope have the greatest effect on the quality of cultivated land, followed by urbanization rate and TAMP.

**Table 5:** LAG model.

Impact Factors	Coefficient	Standard Error	t-Statistics	p-value
**Lagging coverage**	0.390807	0.0532288	5.04154	<0.0001
**Constant**	0.345847	0.0687894	5.02763	<0.0001
**IGR**	0.0175762	0.0124283	-1.4142	0.15730
**Slope**	-0.0429736	0.0168673	-2.54774	0.01084
**Urbanization rate**	-0.00100533	0.000746413	-1.34688	<0.0001
**Pesticide use**	4.83359e-006	5.19545e-006	0.930349	0.17802
**TPOAM**	0.00111366	0.000166644	6.68283	<0.0001

As can be seen in Table 6, the impact of the above-mentioned impact factors on the quality of cultivated land is reflected in the following: (1) For each percentage point increase in IGR, the quality of cultivated land will increase by 2.55%, which shows that the areas with higher quality of cultivated land tend to be distributed along the river, precisely because of the rich water resources, fertile soil and strong cultivated land in the region. (2) For every 1° increase in the slope of the terrain, the quality of cultivated land in Henan Province will be reduced by 8.90%. This is due to the fact that areas with too high slope are not suitable to be reclaimed as cultivated land, and there are clear grading and restrictions on the slope of cultivated land in China. (3) For every 1 percentage point increase in urbanization rate, the quality of cultivated land will be reduced by 0.24%. This result reflects that the damage to the quality of cultivated land and the occupation of cultivated land resources in the process of urbanization and industrial development cannot be ignored, so the protection of cultivated land needs special attention in the future urban planning and development. (4) For every 1 percentage point increase in the total power of agricultural machinery and pesticide use, the quality of cultivated land will be improved by 0.11% and 1.95978e-006 percentage points accordingly. The higher the total power value, the higher the total power of farming machinery in the region, and the higher the quality of cultivated land.

**Table 6:** Error model.

Impact Factors	Coefficient	Standard Error	t-Statistics	p-value
**Constant**	0.673224	0.0484276	13.9017	<0.0001
**IGR**	0.0255177	0.0150107	1.69996	0.08914
**Slope**	-0.0890593	0.0201071	-4.42924	<0.0001
**Urbanization rate**	-0.00238281	0.000769279	-3.09746	0.00195
**Pesticide use**	1.95978e-006	4.58527e-006	0.427408	0.66908
**TPOAM**	0.00112041	0.000156117	7.17672	<0.0001
**LAMBDA**	0.611245	0.0757348	8.07086	<0.0001

**Fig 8:**
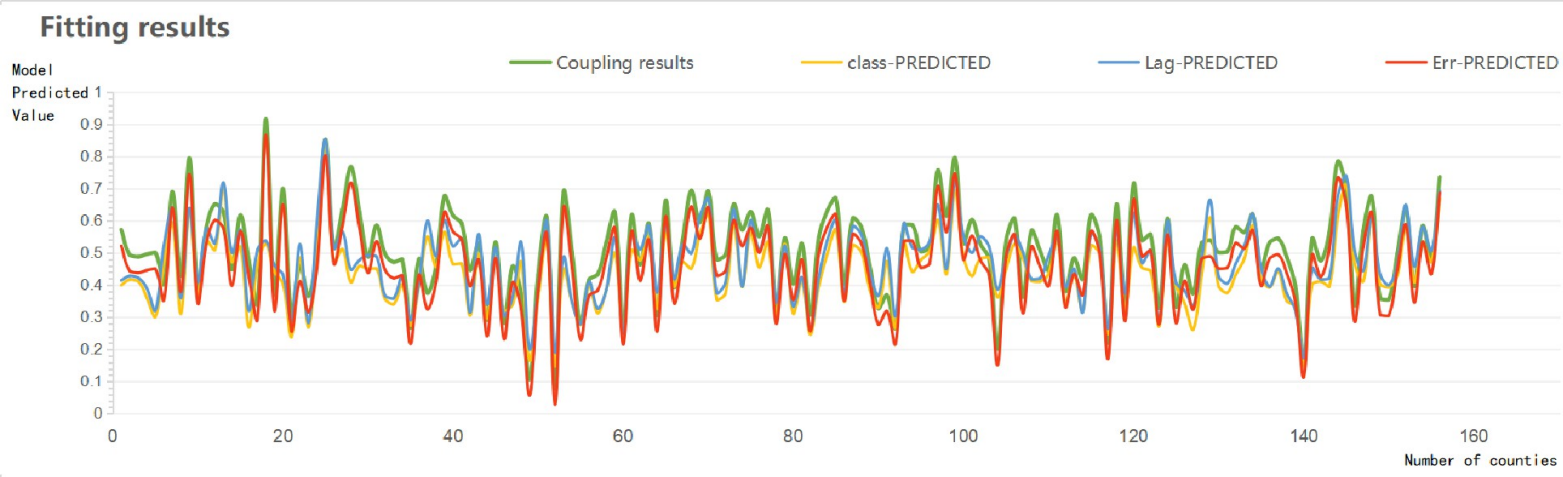
Regression model graph.

At the same time, a fit curve was generated using the actual values of the dependent variable and the predicted values of each regression model, as shown in Fig 8, which shows that the predicted quantity of the spatial err model "Err_PREDIC" fits better with the actual value of "CLQGCA" is better.

### Cultivated land conservation zoning based on local spatial autocorrelation

**Fig 9:**
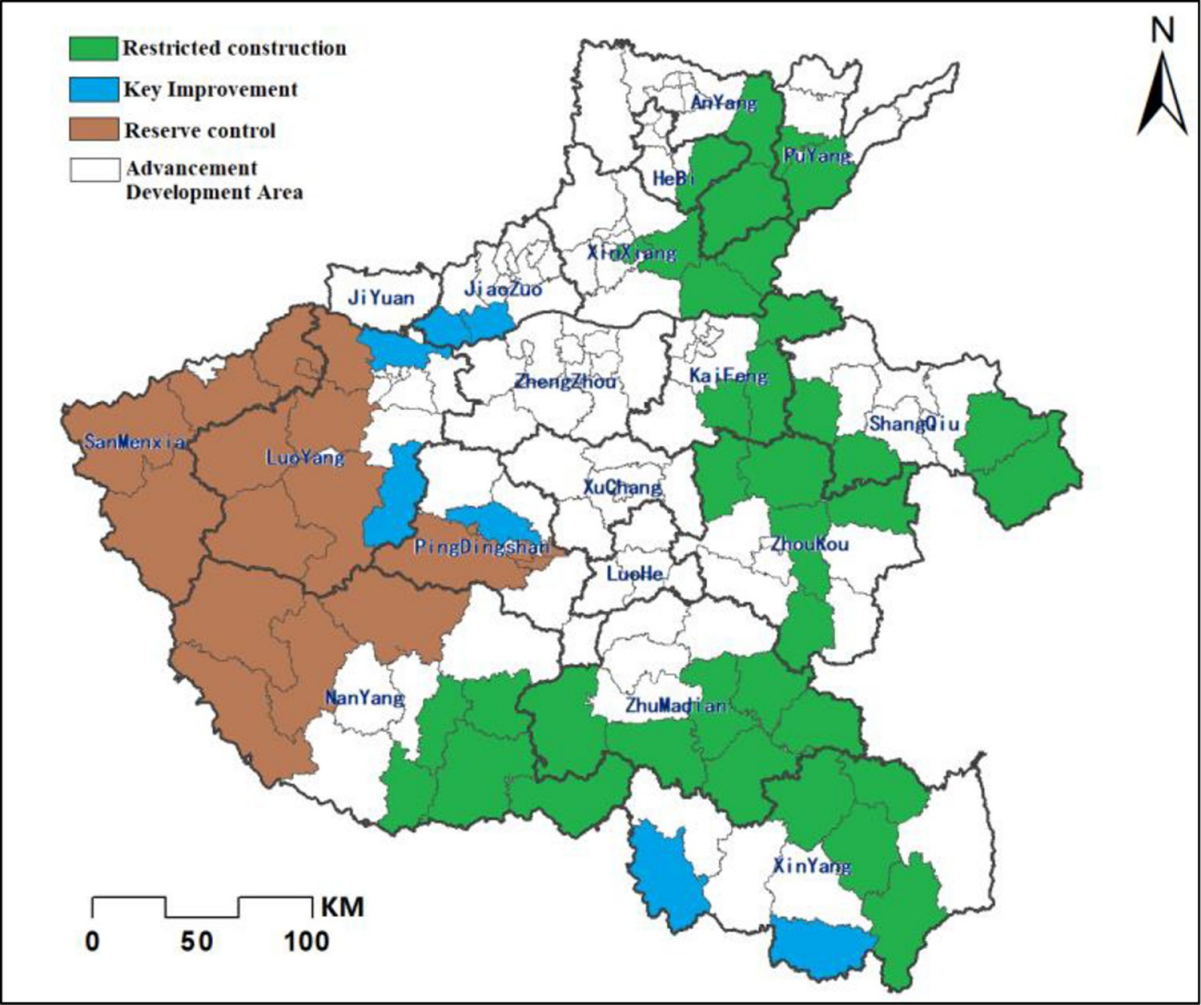
Identification of key protected areas in Henan Province.

Based on the natural quality of cultivated land and considering the spatial correlation and diffusion effect, the conservation of cultivated land in Henan Province is divided into three categories: restricted construction area, reserve control area and key improvement area (as shown in Fig 9).

Among them, the local spatial autocorrelation is HH type cultivated land with high comprehensive quality and obvious aggregation effect. The cultivated land in this area is geographically superior, economically developed, convenient for centralized management, and high natural quality, which is conducive to land rectification. This land area should be designated as a restricted construction zone to strictly prohibit non-agricultural construction. On the contrary, LL type cultivated land is mostly gathered in the western mountainous areas, where the quality of cultivated land, traffic conditions and economic development are relatively poor, making land remediation more difficult. It should be allocated to the reserve control area, based on the basic situation of each county and district to carry out targeted remediation, increase the investment in rectification or reduce fallow for non-agricultural development and construction. The local spatial correlation is LH type, which means that the area of high quality cultivated land is surrounded by the area of low value, due to the influence of spatial polarization. The low value cultivated land in the center is easily assimilated by the surrounding high quality cultivated land. This area has great potential for cultivated land improvement, and should focus on improving the low value area so that the overall transformation to HH type, so this area is designated as a key improvement area. Specific partition results are detailed in Table 7:

**Table 7:** Zoning of cultivated land protection based on local spatial autocorrelation in Henan province.

Cultivated Land quality	Autocorrelation types	Regional name	Typical district	Protection measures	Advice
**CLQGCA**	High-High	Restricted construction	Jiaozuo City, northern Zhengzhou City, Hebi City to Yanjin County, Puyang County and so on	Safeguarding the quality and quantity of currently cultivated land	Prohibiting non-agricultural construction
	Low-Low	Reserve control	Sanmenxia city to Luoyang city, and Nanyang city surrounding counties	Selective rectification according to local conditions	Encourage non-agricultural construction
	Low-High	Key Improvement	Mengjin County and its surrounding counties	Focus on rectifying low-scoring areas	Avoid non-agricultural land development

## Conclusions

Starting from the perspective of spatial pattern, this paper adopts the analysis method of spatial autocorrelation while coupling the normalized results of cultivated land area to study the spatial aggregation characteristics and differences of cultivated land quality in Henan Province at the county level. The spatial autoregressive model was also used to analyze the main driving factors affecting the quality of cultivated land, and finally to propose a more scientific and reasonable zoning protection proposal based on this, with the following conclusions:

The natural quality grades (Moran’s I≈0.71) and CLQGCA (Moran’s I≈0.54), indicating that the quality of cultivated land in Henan Province has a strong correlation in space. The results of the local spatial autocorrelation show that: among the counties with spatial correlation, there are 50 counties with positive correlation type HH and LL, which dominate, and both of them are spatially distributed in aggregation, indicating that the quality attributes of cultivated land in Henan Province are more uniform with spatial attributes.

The results of the bivariate spatial autocorrelation study show that the influence factors in the neighborhood have different degrees of influence on the quality of farmland in Henan Province in space, and there is spatial correlation. Among them, Slope (Moran’s I≈-0.505), IGR (Moran’s I≈0.354), Urbanization rate (Moran’s I≈-0.255), TAMP (Moran’s I≈0.331) and Pesticide use (Moran’s I≈0.214) are the main influencing factors.

From the analysis results of the spatial autoregressive model, we know that: for every 1° increase in slope, the quality of cultivated land in Henan Province will decrease by 8.90%; for every 1 percentage point increase in urbanization rate, the quality of cultivated land will decrease by 0.24%; while for every 1 percentage point increase in TAMP and pesticide use, the quality of cultivated land will increase by 0.11% and 1.95978e-006 percentage points. This indicates that ensuring the quantity and quality of cultivated land in the process of urbanization development cannot be ignored, and increasing the input and construction of agricultural machinery can help guarantee the quantity of land for the construction of planting facilities and improve the quality of cultivated land in the region.

Considering the spatial attributes obtained from the above analysis and the influence of different factors, cultivated land protection in Henan Province is divided into 3 categories: restricted construction area, reserve regulation and control area, and key improvement area. At the same time, the corresponding protection or improvement suggestions are proposed for each area.

Rational spatial optimization of land resources is to achieve sustainable development, of which the allocation of cultivated land resources is the top priority. In addition to improving the quality and protecting the quantity of cultivated land, the focus of research should also be on how to coordinate the relationship between people and land and integrate urban and rural development in the process of urbanization. In addition, global warming nowadays produces a lot of extreme weather, which has a great impact on the quality of cultivated land. Due to the lack of climate data, this study does not include the climate change index as an impact factor, and I will continue to improve this part in future research.
